# Supplementary material for: Gene activation by a CRISPR-assisted trans enhancer
Source: eLife. 2019 Apr 11;8:e45973. doi: 10.7554/eLife.45973 (PMC6478495; doi:10.7554/eLife.45973)
Supplement: Supplementary file 1. [file elife-45973-supp1.docx]

## Supplementary File 1

**Table 1.** Primers were used in high fidelity amplification in this study.

| Name | Sequence |
| --- | --- |
| Lac-px-F | 5′-ACGGTCTCGCACCGGGTCTTCAGCGCCCAATACGCAAACCGCCTC-3′ |
| Lac-px-R | 5′-TCGGTCTCCAAACAGGTCTTCGCGTCCATTCGCCATTCAGGCTGCGC-3′ |
| U6-F | 5′-GAGGGCCTATTTCCCATGATTCC-3′ |
| U6-R | 5′-AAAAAAGCACCGACTCGGTGCCACTTTTTC-3′ |
| U6-1-R | 5′-AAAAAAAAGGTATGTTTGCCGTCGGCCCGATTGCACCGACTCGGTGCCACTTTTTC-3′ |
| U6-2-R | 5′-AAAAAAAAGGTATGTTTGCCGTCGGCCCGATTGCACCGACTCGGTGCCACTTTTTC-3′ |
| U6-3-R | 5′-AAAAAAAAAGGTATGTTTGAGGTTCCACTAGAGCACCGACTCGGTGCCACTTTTTC-3′ |
| dCas9-X-F | 5′-CCGCTCGAGGCCACCATGGACTACAAAGACCATG-3′ |
| dCas9-N-R | 5′-ATAAGAATGCGGCCGCTCAAACTTTGCGTTTCTTTTTCGGGCTAGC-3′ |
| HNF4α-T7-F | 5′-TTCTAATACGACTCACTATAGGGAGCAGGTTGAATTTAGAATGG-3′ |
| HNF4α-P-F | 5′-CCGCTCGAGTGAGATCCAAAACTGAGACAAAAGAAACGGGGCTG-3′ |
| HNF4α-P-R | 5′-CCCAAGCTTAAGCCCACCCAGCCGGAGAGCTGGGGGCATGGAGG-3′ |
| HNF4α-sP-F | 5′-tagtaggtgctcaataaatatgttag-3′ |
| HNF4α-sP-R | 5′-GGCTTTACCAACAGTACCGGATCT-3′ |
| CMV-F | 5′-TAGTTATTAATAGTAATCAATTACGGGG-3′ |
| CMV-1-R | 5′-AATCGGGCCGACGGCAAACATACCCCTCAGCAGCTCTGCTTATATAGACCTCCCACC-3′ |
| CMV-2-R | 5′-GGAACCTTACGAATACCAGATGCTCCTCAGCAGCTCTGCTTATATAGACCTCCCACC-3′ |
| CMV-3-R | 5′-ATCTAGTGGAACCTCAAACATACCCCTCAGCAGCTCTGCTTATATAGACCTCCCACC-3′ |
| CS-1-R | 5′-GGGGTATGTTTGCCGTCGGCCCGATT-3′ |
| CS-2-R | 5′-GGAGCATCTGGTATTCGTAAGGTTCC-3′ |
| CS-3-R | 5′-GGGGTATGTTTGAGGTTCCACTAGAT-3′ |
| EF1-a-F | 5′-GCTCCGGTGCCCGTCAGTGGGCAGAGC-3′ |
| EF1-a-R | 5′-GGAACCTTACGAATACCAGATGCTCCTCAGCTCACGACACCTGAAATGGAAGAAAA-3′ |
| PGK-F | 5′-GGGTAGGGGAGGCGCTTTTCCCAAGGCAG-3′ |
| PGK-R | 5′-GGAACCTTACGAATACCAGATGCTCCTCAGCCGAAAGGCCCGGAGATGAGGAAG-3′ |
| GAL4-BsiEW-F | 5′-TACCCGTACGGAATGAAGCTACTGTCTTCTATCGAACAAG-3′ |
| GAL4-BspE-R | 5′-GTAGTCCGGATTACGATACAGTCAACTGTC-3′ |
| 5×UAS | 5′-CTCAGATCTCGAGCTCAAGCTTCGAACGGTCAACAGTTGTCCGCGGTCAACAGTTGTCCGCGGTCAACAGTTGTCCGCGGTCAACAGTTGTCCGCGGTCAACAGTTGTCCG-3′ |
| UAS-CMV- Bgl-F | 5′-CGTTACATAACTTACGGTAAATGGCCCGCCTG-3′ |
| UAS-CMV- Hind-R | 5′-TTCGAAGCTTAGCTCTGCTTATATAGACCTCC-3′ |
| UAS-CMV-R | 5′-CACTATAGGGCGAATTGAAGCTGCCCTT-3′ |

**Table 2.** Oligonucleotide used to prepare target-specific regions (20 bp) of sgRNA

| Gene | Name | Sequence |
| --- | --- | --- |
| HNF4α | HNF4α-sg-S | 5′-AGGAAGACGGCACCGGCCAGTCACTTAGGGAACCCGGTTTGGGTCTTCGA-3′ |
|  | HNF4α-sg-AS | 5′-TCGAAGACCCAAACCGGGTTCCCTAAGTGACTGGCCGGTGCCGTCTTCCT-3′ |
| E47 ( TCF3) | TCF3-sg-S | 5′-AGGAAGACGGCACCGGGCGCACGGGCCCCGCGGGACGTTTGGGTCTTCGA-3′ |
|  | TCF3-sg-AS | 5′-TCGAAGACCCAAACGTCCCGCGGGGCCCGTGCGCCCGGTGCCGTCTTCCT-3′ |
| ASCL1 | ASCL1-sg-S | 5′-AGGAAGACGGCACCGGAGCCGCTCGCTGCAGCAGCGGTTTGGGTCTTCGA-3′ |
|  | ASCL1-sg-AS | 5′-TCGAAGACCCAAACCGCTGCTGCAGCGAGCGGCTCCGGTGCCGTCTTCCT-3′ |
| Ngn2 | Ngn2-sg-S | 5′-AGGAAGACGGCACCGGCTGACAGGAGGAGGAGGCGGGTTTGGGTCTTCGA-3′ |
|  | Ngn2-sg-AS | 5′-TCGAAGACCCAAACCCGCCTCCTCCTCCTGTCAGCCGGTGCCGTCTTCCT-3′ |
| Oct4 | Oct4-sg-S | 5′-AGGAAGACGGCACCGGGGAAAACCGGGAGACACAACGTTTGGGTCTTCGA-3′ |
|  | Oct4-sg-AS | 5′-TCGAAGACCCAAACGTTGTGTCTCCCGGTTTTCCCCGGTGCCGTCTTCCT-3′ |
| Nanog | Nanog-sg-S | 5′-AGGAAGACGGCACCGGAGAGTAACCCAGACTAGGTGGTTTGGGTCTTCGA-3′ |
|  | Nanog-sg-AS | 5′-TCGAAGACCCAAACCACCTAGTCTGGGTTACTCTCCGGTGCCGTCTTCCT-3′ |
| TNFAIP3 | TNFAIP3-sg-S | 5′-AGGAAGACGGCACCGGCCGCCCCGCCCGGTCCCTGCGTTTGGGTCTTCGA-3′ |
|  | TNFAIP3-sg-AS | 5′-TCGAAGACCCAAACGCAGGGACCGGGCGGGGCGGCCGGTGCCGTCTTCCT-3′ |
| CASP9 | CASP9-sg-S | 5′-AGGAAGACGGCACCGGGGTGACGTGAGGTCAGTGCGGTTTGGGTCTTCGA-3′ |
|  | CASP9-sg-AS | 5′-TCGAAGACCCAAACCGCACTGACCTCACGTCACCCCGGTGCCGTCTTCCT-3′ |
| CSF3 | CSF3-sg-S | 5′-AGGAAGACGGCACCGGGGGAGGAAGGGAGTTTGAGGGTTTGGGTCTTCGA-3′ |
|  | CSF3-sg-AS | 5′-TCGAAGACCCAAACCCTCAAACTCCCTTCCTCCCCCGGTGCCGTCTTCCT-3′ |
| Sox2 | Sox2-sg-S | 5′-AGGAAGACGGCACCGGGGAGGAGGGGGCAGGCGAGGGTTTGGGTCTTCGA-3′ |
|  | Sox2-sg-AS | 5′-TCGAAGACCCAAACCCTCGCCTGCCCCCTCCTCCCCGGTGCCGTCTTCCT-3′ |

S, sense; AS, antisense.

**Table 3.** Primers used as qPCR detection of gene expression (see Figure 5).

| Gene | Name | Sequence |
| --- | --- | --- |
| Hnf4a | Hnf4a-F | 5′-GACATTCGGGCGAAGAAGAT-3′ |
|  | Hnf4a-R | 5′-AAGATGATGGCTTTGAGGTAGG-3′ |
| TNFAIP3 | TNFAIP3-F | 5′-TCATCCACAAAGCCCTCATC-3′ |
|  | TNFAIP3-R | 5′-CATCATTCCAGTTCCGAGTATCA-3′ |
| TCF3 | TCF3-F | 5′-ACGAGCGTATGGGCTACCA-3′ |
|  | TCF3-R | 5′-GTTATTGCTTGAGTGATCCGGG-3′ |
| CSF3 | CSF3-F | 5′-CCGACTCCTACAGTGGGCTA-3′ |
|  | CSF3-R | 5′-CGCTGACGTGTTCTCCTCG-3′ |
| ASCL1 | ASCL1-F | 5′-CGCGGCCAACAAGAAGATG-3′ |
|  | ASCL1-R | 5′-CGACGAGTAGGATGAGACCG-3′ |
| Ngn2 | Ngn2-F | 5′-AGGAAGAGGACGTGTTAGTGC-3′ |
|  | Ngn2-R | 5′-GCAATCGTGTACCAGACCCAG-3′ |
| Sox2 | Sox2-F | 5′-GTGAACCAGCGCATGGACAG-3′ |
|  | Sox2-R | 5′-TCTGCGAGCTGGTCATGGAG-3′ |
| Oct4 | Oct4-F | 5′-CTTGAATCCCGAATGGAAAGGG-3′ |
|  | Oct4-R | 5′-GTGTATATCCCAGGGTGATCCTC-3′ |
| Nanog | Nanog-F | 5′-CCTGGAACAGTCCCTTCTATAAC-3′ |
|  | Nanog-R | 5′-TCACTCATCTTCACACGTCTTC-3′ |
| CASP9 | CASP9-F | 5′-CGAACTAACAGGCAAGCAGCAAAG-3′ |
|  | CASP9-R | 5′-AGAGCACCGACATCACCAAATCC-3′ |
| GAPDH | GAPDH-F | 5′-GGATTTGGTCGTATTGGG-3′ |
|  | GAPDH-R | 5′-GGAAGATGGTGATGGGATT-3′ |

**Supplementary Table 4.** Primers used as qPCR detection of gene expression (see Figure 6).

| Gene | Name | Sequence |
| --- | --- | --- |
| CD90 | CD90-F | 5′-CGGAAGACCCCAGTCCA-3′ |
|  | CD90-R | 5′-ACGAAGGCTCTGGTCCACTA-3′ |
| CD133 | CD133-F | 5′-ACATGAAAAGACCTGGGGG-3′ |
|  | CD133-R | 5′-GATCTGGTGT CCCAGCATG-3′ |
| Oct4 | Oct4-F | 5′-CTTGAATCCCGAATGGAAAGGG-3′ |
|  | Oct4-R | 5′-GTGTATATCCCAGGGTGATCCTC-3′ |
| Sox2 | Sox2-F | 5′-GTGAACCAGCGCATGGACAG-3′ |
|  | Sox2-R | 5′-TCTGCGAGCTGGTCATGGAG-3′ |
| Nanog | Nanog-F | 5′-CCTGGAACAGTCCCTTCTATAAC-3′ |
|  | Nanog-R | 5′-TCACTCATCTTCACACGTCTTC-3′ |
| LIN28 | LIN28-F | 5′-TGTAAGTGGTTCAACGTGCG-3′ |
|  | LIN28-R | 5′-CCTCACCCTCCTTCAAGCTC-3′ |
| c-Myc | c-Myc-F | 5′-GCTGCTTAGACGCTGGATTT-3′ |
|  | c-Myc-R | 5′-CTCCTCCTCGTCGCAGTAGA-3′ |
| Klf4 | Klf4-F | 5′-GCGGCAAAACCTACACAAAG-3′ |
|  | Klf4-R | 5′-CCCCGTGTGTTTACGGTAGT-3′ |
| P21 | P21-F | 5′-GGATGTCCGTCAGAACCCAT-3′ |
|  | P21-R | 5′-CCCTCCAGTGGTGTCTCGGTG-3′ |
| GS | GS-F | 5′-CCTGCTTGTATGCTGGAGTC-3′ |
|  | GS-R | 5′-GAAAAGTCGTTGATGTTGGA-3′ |
| BR | BR-F | 5′-ACAAGGTGCTGCGGGAATCA-3′ |
|  | BR-R | 5′-ACTGGTGGGAGGGGTAGGTG-3′ |
| ALDOB | ALDOB-F | 5′-AGGAGGACTCTTCTCTCCCAA-3′ |
|  | ALDOB-R | 5′-GATTCATCTGCAGCCAGGAT-3′ |
| CYP1α2 | CYP1α2-F | 5′-CTGGCCTCTGCCATCTTCTG-3′ |
|  | CYP1α2-R | 5′-TTAGCCTCCTTGCTCACATGC-3′ |
| PEPCK | PEPCK-F | 5′-GTGTCCCTCTAGTCTATGAAGC-3′ |
|  | PEPCK-R | 5′-ATTGACTTGATCCTCCAGATAC-3′ |
| APOCIII | APOCIII-F | 5′-GGGTACTCCTTGTTGTTGC-3′ |
|  | APOCIII-R | 5′-AAATCCCAGAACTCAGAGAAC-3′ |
| G-6-P | G-6-P-F | 5′-GGCTCCATGACTGTGGGATC-3′ |
|  | G-6-P-R | 5′-TTCAGCTGCACAGCCCAGAA-3′ |
| CPA2 | CPA2-F | 5′-AGTGGGTTACACAAGCTACG-3′ |
|  | CPA2-R | 5′-AGAGGCTTCCAGATACCTTG-3′ |
| CX32 | CX32-F | 5′-CTGCAGACATTCTCTGGGAAA-3′ |
|  | CX32-R | 5′-GCACCATGATTCTGAAGATGA-3′ |
| TP53INP1 | TP53INP1-F | 5′-AAACCTTCTCATTGAACATCCC-3′ |
|  | TP53INP1-R | 5′-CCATTGTGCTTGACTTGCC-3′ |
| MIST1 | MIST1-F | 5′-CCAGCACTACCAGCAGCA-3′ |
|  | MIST1-R | 5′-AGGACTGGGCGCTAGGTG-3′ |
| PRSS2 | PRSS2-F | 5′-GTTGCAGCTGCTGTTGCTGCC-3′ |
|  | PRSS2-R | 5′-TGTCATTGTCCAGAGTCCGGC-3′ |
| CELA3A | CELA3A-F | 5′-CTCGTGTGTGGCCTGTGGCA-3′ |
|  | CELA3A-R | 5′-CCAGAAACAAAGCTGGTCACA-3′ |
