# Supplementary figures and images for: Gene activation by a CRISPR-assisted trans enhancer
Source: eLife. 2019 Apr 11;8:e45973. doi: 10.7554/eLife.45973 (PMC6478495; doi:10.7554/eLife.45973)

## Supplementary File 2


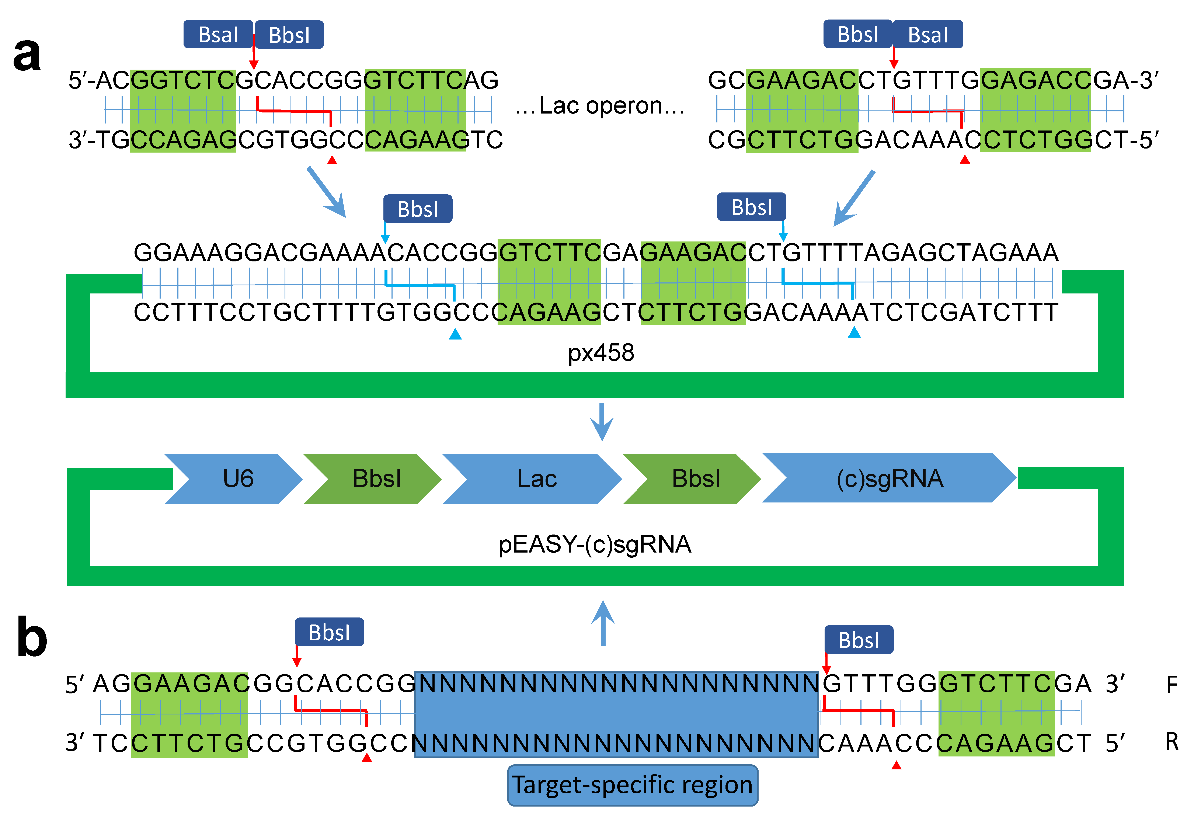


Schematic show of construction of sgRNA vectors for blue-white screening.

Supplement: Supplementary file 2. [file elife-45973-supp2.docx]
